# Warum in Königsberg, warum Samuel Jessner, warum 1921? Hintergründe zum ersten universitären Lehrauftrag für Sexualwissenschaft in Deutschland

**DOI:** 10.1007/s00120-021-01611-8

**Published:** 2021-08-25

**Authors:** Friedrich H. Moll, Richard Kühl, Matthis Krischel, Thorsten Halling, Heiner Fangerau

**Affiliations:** 1 Institut für Geschichte, Theorie und Ethik der Medizin, Centre for Health and Society, Medizinische Fakultät, Heinrich-Heine-Universität, Düsseldorf, Deutschland; 2 Curator Museum, Bibliothek und Archiv, Deutsche Gesellschaft für Urologie e. V., Düsseldorf – Berlin, Deutschland; 3 Urologische Klinik, Urologischer Arbeitsplatz Krankenhaus Merheim, Kliniken der Stadt Köln gGmbH, Neufelder Straße 32, 51967 Köln, Deutschland

**Keywords:** Geschichte der Urologie, Wissenschaftsgeschichte, Etablierung von Lehrstühlen, History of urology, History of science, Establishment of lectureships

## Abstract

Der Dermatovenerologe Samuel Jessner (1859–1929) erhielt im Jahr 1921 einen Lehrauftrag für „Sexuallehren“ an der Universität Königsberg (heute: russ Калининград, Kaliningrad). Im Reichsmedizinalkalender wurde er aber auch als Urologe geführt. Dieser Beitrag zeichnet seinen Lebensweg und sein Wirken nach und fragt, wie Jessner in der Peripherie der deutschen Sexualwissenschaft und ohne enge Anbindung an deren Netzwerke dieser akademische Erfolg gelingen konnte. Sein geringer Einfluss in der Forschung, seine fehlende Anbindung an eine „Schule“ der Sexualwissenschaft im deutschsprachigen Raum und seine jüdische Herkunft waren Faktoren, die sowohl die Wahrnehmung seiner Arbeit unter Zeitgenossen als auch das Vergessen seiner Tätigkeiten in der Geschichtsschreibung präformierten.

## Abstract (en)

The dermatologist and venerologist Samuel Jessner (1895–1929) received a lectureship for sexology at the University of Koenigsberg (today: Russian Калининград, Kaliningrad) in 1921. Since 1928 he was also listed as a urologist in the *Reichsmedizinalkalender* (German Physician Address Calendar). In this article we trace his life and work and ask how Jessner was able to achieve this academic success in the periphery of German sexology and without close ties to its networks. His weak influence in research, his lack of connection to a “school” of sexual science in German-speaking countries, and his Jewish origin were factors that impaired both the recognition of his work among his contemporaries and his recognition in the discipline-specific historiography until today.

## Einleitung

Im Jahr 1921 erteilte die Universität Königsberg einen Lehrauftrag für Sexualwissenschaft („Sexuallehre“). Das war nicht nur im deutschsprachigen Raum ein Novum. Soweit bekannt, hatte sich bis dahin keine Universität weltweit zu einem solchen Schritt entschließen können. In Königsberg wurde der Dermatovenerologe Samuel Jessner (1859–1929) mit der Aufgabe betraut, das Fach in seiner ganzen Breite zu vertreten. Mit Jessner erhielt die Sexualforschung in Königsberg zwar eine Berücksichtigung in der akademischen Lehre. Auch zeigt seine Bioergographie, dass Dermatovenerologen als Spezialisten für Geschlechtskrankheiten wichtige Rollen in der sich entwickelnden Sexualwissenschaft – hier den ersten formalen universitären Lehrauftrag – einnehmen konnten. Gleichzeitig blieb Jessner aber langfristig ohne viel Einfluss. Nach seinem Tod geriet er bald in Vergessenheit. Dieser Beitrag ordnet Jessner und seine Position in Königsberg auf der Basis der Sekundärliteratur und bisher nicht beachteter Quellen in die Geschichte der Sexualwissenschaft im deutschsprachigen Raum ein und fragt, welche Umstände es vielleicht beförderten, dass er ohne eine enge Anbindung an die bestehenden Netzwerke in den deutschsprachigen „Wissenschaftshauptstädten“ Berlin und Wien diesen ersten Lehrauftrag erhalten konnte.

## Der weltweit erste Lehrauftrag für Sexualwissenschaft

**Figure 1:**
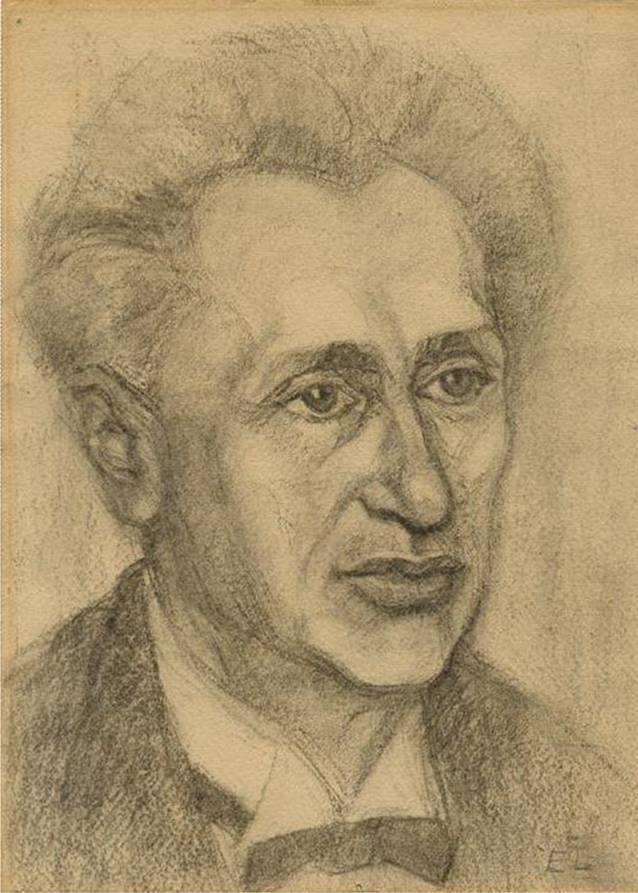

Im Jahr 1921 machte nicht nur in der medizinischen Presse^1^, sondern auch in sexualwissenschaftlichen Foren [1] die Meldung die Runde, dass für den Arzt Samuel Jessner (Abb. 1) an der Universität Königsberg erstmals in Deutschland ein Lehrstuhl oder eine Professur für Sexualwissenschaft („Sexuallehre“) eingerichtet worden sei. Auch auf dem im selben Jahr abgehaltenen I. Internationalen Kongress für Sexualreform in Berlin, veranstaltet vom 1919 von Magnus Hirschfeld (1868–1935; [2]) als Privatstiftung gegründeten Institut für Sexualwissenschaft, wurde Jessner in den Eröffnungsreden in dieser Funktion vorgestellt [3]. Seitdem findet sich diese Angabe in den Annalen der Fachgeschichte, auch wenn sie nicht ganz zutrifft: Der Hirschfeld-Biograf Manfred Herzer hat im Jahr 2017 wohl als erster unzweifelhaft verifiziert, dass es sich bei Jessners Position nicht um einen Lehrstuhl, sondern um einen unbezahlten Lehrauftrag (ohne Extraordinariat) gehandelt hatte [4]. Dennoch stellte dieser vor genau einem Jahrhundert erteilte Lehrauftrag ein historisches Ereignis dar. Pioniere wie Albert Eulenburg (1840–1917) hatten zwar schon im Kaiserreich Vorlesungen gehalten, die als rein sexualwissenschaftliche Formate hätten gelten können [5]. Doch eine offizielle, sogar dauerhafte Vergabe eines Lehrauftrags für das Fachgebiet hatte es zuvor, soweit bekannt, an keiner Universität weltweit gegeben.

Über seinen Inhaber, Samuel Jessner, war bisher, wie der Sexualforscher Volkmar Sigusch in einem biografisch tastenden Artikel für das 2009 erschienene „Personenlexikon der Sexualforschung“ zutreffend geschrieben hat, erstaunlich wenig in Erfahrung zu bringen [6]. Dasselbe gilt nach wie vor für eine Einordung seines wissenschaftlichen Werks.

## Biografische Skizze

Laut verschiedener biografischer Lexika wurde Samuel Jessner^2^ entweder in Berlin oder Königsberg geboren. Beides ist falsch. Jessner kam am 5. Januar 1859 [7] im heute litauischen Darbėnai (russ. Dorbyany Дорбяны, Kreis Tels[c]hi [lit. Telšiai; dt. „Telz“], Gouvernemet Kovno Ковенcкая губерния/Kowenskaja gubernija im „Russischen Reich“, dt. „Dorben“ „Drobian/Dorbian in Großlittauen“, Dorbian 
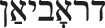
 [Yid], Dorbiany [Pol], Darbian, Darbėnų, Darbienā, Darbenay, Durbiany) zur Welt., ca. 45 km von Memel (heute lit. Klaipeda) – bis 1919 die nördlichste Stadt des Deutschen Reiches – entfernt [8]. In der Region hatten Deutsche, Litauer und Russen über die Jahrhunderte hinweg in verschiedenen Staatssystemen gelebt [9]. Ein Großteil der Bevölkerung der Stadt war jüdischer Konfession oder Herkunft. Im Jahre 1900 lag ihr Anteil bei rund 80% [10].^3^

Soweit ersichtlich, findet sich die erste Fehlangabe 1901 in dem bekannten Ärztelexikon von Julius Pagel (1851–1912; [11]), der seine Informationen wahrscheinlich von den erfassten Personen direkt eingeholt hatte, was die Möglichkeit offen lässt, dass eine der fehlerhaften Angaben von Jessner selbst stammte. Mögliche, auch sehr naheliegende Gründe, die eigene Herkunft ins damalige Preußen zu rücken, können antisemitische Diskriminierungserfahrungen, insbesondere osteuropäischen Juden gegenüber, gewesen sein.

Samuel Jessner scheint etwa 5 Jahre alt gewesen sein, als er nach Königsberg kam. Der Vater, der einer kaufmännischer Tätigkeit nachging, war mit der Familie im Jahre 1863 oder 1864 in die rund 260 km entfernte Großstadt in Ostpreußen umgesiedelt, vermutlich, weil die wirtschaftlich prosperierende Metropole bessere Berufs- und Ausbildungsmöglichkeiten für seine Kinder bot als das russisch-litauische Schtetl [12].^4^,^5^

Seine gymnasiale Ausbildung erhielt Samuel Jessner am Königsberger Altstädtischen Gymnasium, einer traditionsreichen Einrichtung, die von den Söhnen des sozial höhergestellten jüdischen Königsberger Bürgertums besucht wurde [13]. Hier legte er Ostern 1876 im Alter von 17 Jahren die Reifeprüfung ab. Nach dem erfolgreichen Studium der Medizin an der Albertina in Königsberg von 1876 bis 1881 wurde er am 21. Mai 1881 in Leipzig [14–16] promoviert. Bereits 1880, als Assistent des Physiologen William Alfred Grünhagen (1842–1912) trat Jessner mit einer Zeitschriftenpublikation hervor [17].

Noch 1881 zog Jessner nach Stolp (Stolpmünde) in Hinterpommern (heute: Słupsk/Polen). Spätestens dort heiratete er Helene Eliascheff. Das erste Kind des Ehepaars, Tochter Elsa „Ellon“ (1885–um 1960), wurde dort geboren, ebenso wie zwei der insgesamt drei Söhne, die ohne Ausnahme in ihren Berufen Bekanntheit erlangen sollten: Einmal der Dermatovenerologe Kurt Joachim Jessner (1886–1951 Maine) und dessen 1926 zum außerordentlichen Professor für Dermatologie in Breslau ernannter Bruder Max (1887–1978 Schweiz)^6^, sowie Fritz (1889–1946), der Theaterschauspieler wurde, zeitweilig Intendant am Neuen Schauspielhaus in Königsberg war und nach Emigration im Jahr 1940 in die „Yale School of Drama“ eintrat [20].

Zur Schule gegangen waren die Kinder alle nicht mehr in Stolp, wo Samuel Jessner zuletzt als „praktischer Arzt“ in der Bahnhofstraße 43 gemeldet war [21, 22], sondern in Königsberg, wo Jessner sich – wahrscheinlich 1891 – als Dermatologe niedergelassen hatte.

## Jessners biografische Brücken zur Sexualwissenschaft

**Figure 2:**
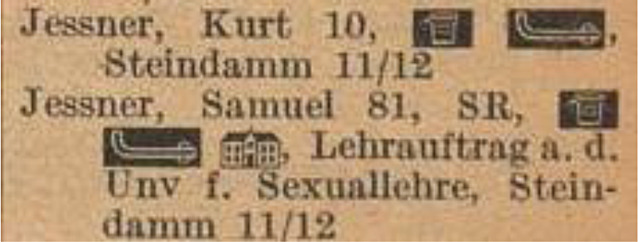

**Figure 3:**
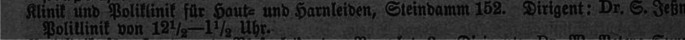

In seiner Königsberger Praxis dürften frühzeitig sexualmedizinisch relevante Leiden behandelt worden sein. Unmittelbar vor der Rückkehr in die Stadt, in der er aufgewachsen war, gab Jessner an, „Vorstudien in Dermato-Syphilidologie“ [14] absolviert zu haben [23]. Und ausweislich seiner späteren medizinischen Publikationen darf er als Urologe *avant la lettre*, also vor der Einführung des Facharztes in Deutschland 1924 [24], gelten [25]. 1894 schließlich wurde Jessner, der allerdings nie Mitglied der Deutschen Gesellschaft für Urologie werden sollte, in die Deutsche Dermatologische Gesellschaft aufgenommen [26, 27]. Im Reichsmedizinalkalender von 1927 erscheint Samuel Jessner – ebenso wie sein Sohn Kurt – mit den Symbolen für Dermatologie-Venerologie und für Urologie (Abb. 2). Dies weist auf die Unschärfe zwischen den Tätigkeitsfeldern der Spezialärzte für Haut‑, Harn-, und Geschlechtsleiden zu dieser Zeit hin. Bereits im Jahr 1899 hatte Jessner im Adressbuch für Königsberg als „Dirigent“ einer „Klinik und Poliklinik für Haut und Harnleiden“ firmiert (vgl. Abb. 3). Dies kann durch die Überschneidung der Bereiche erklärt werden, vielleicht aber auch als Euphemismus für eine Praxis verstanden werden, die sich auf die Behandlung von Geschlechtskrankheiten spezialisiert hatte.

Zugleich war es nicht zuletzt diese doppelte Schwerpunktlegung, die Jessner bald auch in die Sexualwissenschaft im engeren Sinne führen sollte. Das galt nicht nur für ihn: Von den Haut- und Geschlechtskrankheiten führten viele Wege in die damals noch junge, sich erst um 1905 als eigenständig formierende Fachdisziplin. Denn auch wenn sich die frühe Sexualwissenschaft äußerst interdisziplinär aufstellte (und dabei keineswegs auf die Medizin beschränkt war), lieferten doch eine ganze Reihe von Dermatovenerologen bedeutende Beiträge zur Konstituierung des Faches, darunter, der schon zeitgenössisch berühmte Iwan Bloch (1872–1922) aus Berlin, einem Zentrum der damaligen Sexualwissenschaft [28–30].

Jessner engagierte sich auch in der Deutschen Gesellschaft für ethische Kultur, was ihm vermutlich ebenfalls eine wichtige Brücke zur frühen Sexualwissenschaft schlug. Ähnlich wie in der monistischen Bewegung, die in Deutschland eine große Anziehung auf die Sexualreformbewegung ausübte [31], versammelten sich in den Strukturen der internationalen Ethikkulturbewegung aus verschiedenen wissenschaftlichen Disziplinen stammende Vertreterinnen und Vertreter. Sie forderten konkrete gesellschaftspolitische Veränderungen wie die Einführung des Frauenstimmrechts und hatten eine mehr oder weniger klar umrissene sexual- und sozialethische Zukunftsprogrammatik. Der innerhalb der Sexualforschung wohl wichtigste Vertreter dieser heute fast vergessenen Richtung war der aus Königsberg stammende Kunsthistoriker und Pädagoge Bruno Meyer (1840–1917), der sich im „Bund für Mutterschutz“ engagierte und 1908 in den Gründungsstab der ständigen Mitarbeiter von Max Marcuses (1877–1963 Tel Aviv) Zeitschrift *Sexual-Probleme* eintrat.^7^

## Aus der sexualmedizinischen Praxis an die Universität

Auch wenn sich also aus Jessners Tätigkeiten Bezüge zur Sexualwissenschaft ableiten lassen, die seinen Antrag auf Erteilung eines Lehrauftrags für „Sexuallehren“ begründen können, konstatierte die Universität Königsberg dem preußischen Kultusministerium gegenüber am 24. Juni 1921, dass „*Dr. Jessner auf diesem Gebiete bisher nicht merklich hervorgetreten ist*“^8^. Wie lässt es sich also erklären, dass Jessner einen solchen Lehrauftrag beantragte, und wie ist zu erklären, dass dieser kurz nach der wenig enthusiastischen Aussage der Universität tatsächlich erteilt wurde?

Anfang der 1920er-Jahre gehörte seine Praxis fraglos zu den sexualmedizinisch prominenten Adressen, in die zu dieser Zeit bereits einer der beiden Söhne eingetreten war: Kurt Jessner, um 1920 kurzzeitig Assistent seines Vaters, hatte zuvor bei dem berühmten Dermatologen und Sexualforscher Albert Neisser (1855–1916; [33]) in Breslau gearbeitet.^9^ Sein Bruder Max Jessner zuletzt führte die Praxis bis zu seiner Emigration 1935 weiter.

In wissenschaftlicher Hinsicht hingegen hatte Samuel Jessner bis dahin keine nennenswerten Spuren in der Fachgeschichte der Sexualwissenschaft/Sexualmedizin hinterlassen. Das gilt darüber hinaus auch für seine Präsenz in ihren Strukturen. Weder war er Mitglied einer der bestehenden Fachgesellschaften, z. B. der „Ärztlichen Gesellschaft für Sexualwissenschaft und Eugenik“ oder der „Internationalen Gesellschaft für Sexualforschung“ (beide gegründet 1913), noch hatte er in den einschlägigen Fachforen wie der *Zeitschrift für Sexualwissenschaft* publiziert. Auch sonstige Kooperationen mit frühen Proponenten der Sexualwissenschaft oder deren Institutionen lassen sich bis dahin nicht belegen.

Irgendwelche Einwände gegen Erteilung des Lehrauftrags, so die Fakultät über Jessner, bestünden jedoch nicht, zumal der Lehrauftrag unbesoldet und jederzeit widerrufbar erteilt wurde. Mit Schreiben vom 12. Juli 1921 wurde Jessner also mitgeteilt, dass er vom Wintersemester 1921/1922 an einen unbezahlten Lehrauftrag an der Albertina erhalte, um „die Sexuallehre in Vorlesungen und soweit nötig in Übungen zu vertreten“^10^. Damit kamen Universität und Ministerium ohne Einschränkung seinem Wunsch nach, dieses Fach „vom biologischen, medizinisch-hygienischen, pädagogischen, ethischen Standpunkte aus“ dem akademischen Nachwuchs nahebringen zu dürfen^11^.

## Eine Einordung in die Geschichte der Sexualwissenschaft in Deutschland

Es spricht vieles dafür, in diesem Zugeständnis der Universität Königsberg einen Ausdruck dessen zu erblicken, was Laurie Marhoefer als „Weimar settlement on sexual politics“ gefasst hat [34]. Demnach war, wie die Historikerin dies am Beispiel der Wahrnehmung der Homosexualität nachvollzogen hat, nach 1918 sexuellen Themen eine gewisse Relevanz tolerierend eingeräumt worden, solange diese in der Öffentlichkeit nicht zu deutlich sichtbar wurden. Jessner verkörperte diese prekäre Akzeptanz mit Blick auf die Sexualwissenschaft an deutschen Universitäten auf mustergültige, weil harmlose Weise.

In diese Richtung deuten historische Vergleiche fast zwingend. Im Unterschied zu Jessner hatte etwa der immer wieder mit dem berühmten Berliner Sexualreformer Magnus Hirschfeld (1868–1935) in Kooperation getretene Leipziger Sexualmediziner Hermann Rohleder (1866–1934) 1923 von der Universität Leipzig eine rüde Abfuhr erhalten, als er sich dort um einen Lehrauftrag für Sexualwissenschaft bemüht hatte [35]. Rohleder konnte zu diesem Zeitpunkt bereits auf ein – im Vergleich zu Jessner fachlich ungleich breites, ja im Grunde universalsexologisches – Wirken zurückblicken [36]. Eine ähnliche Zurückweisung hatte auch schon zuvor der international renommierte Dermatovenerologe Alfred Blaschko (1858–1922) erfahren, dessen sexualreformerisches Engagement sich vor allem auf das Feld der Prostitution erstreckte. Als für Blaschko an der Universität Berlin kurz nach der Revolution von 1918/19 ein Lehrstuhl eingerichtet werden sollte, verhinderte eine ehrabschneidende Stellungnahme der dortigen Medizinischen Fakultät jede Perspektive auf eine universitäre Anerkennung seiner Lebensarbeit [37]. Ein nicht minder für sich sprechendes Bild bot die Universität Jena, als sie nach dem Tod des Stiftungsgebers Hans Holbein (1864–1929) das bereits angenommene Stiftungskapital für eine sexualwissenschaftliche Vorlesung einem Labor der universitätseigenen Pathologie zukommen ließ. Holbein, der sich zu Lebzeiten in Magnus Hirschfelds Wissenschaftlich-humanitären Komitee für die Abschaffung des Antihomosexuellenparagraphen 175 eingesetzt hatte, wollte mit seiner Stiftung eine öffentliche Vorlesung zum Thema Homo- oder Bisexualität verwirklicht gesehen haben [38, 39].

Samuel Jessner dagegen bot keinerlei vergleichbare Angriffsflächen, hielt sich von sexualreformerischen Verbänden fern, stand z. B. mit Helene Stöcker (1869–1943 New York) und dem „Bund für Mutterschutz“ in keinerlei Verbindung, und auch der Kontakt zu Magnus Hirschfeld und dessen Institut für Sexualwissenschaft in Berlin war eher lose. Darüber mag hinweggetäuscht haben, dass Jessner auf dem I. Internationalen Kongress für Sexualreform auf sexualwissenschaftlicher Grundlage in Berlin so prominent begrüßt wurde und er offenbar noch dem Ausschusses für die Planung des zweiten Kongresses 1922 in Rom angehörte, der allerdings nicht stattfand [40]. Sein Name taucht auch in den Gremien der Hirschfeld-nahen „Weltliga für Sexualreform“ nicht auf, die mit dem Ziel nicht nur der Verstetigung internationaler Tagungen, sondern auch zur Formulierung einer transnational wirksamen Programmatik gegründet worden war und die damit zumindest bis 1930, als ihr vierter Kongress medial vielbeachtet in Wien stattfand, äußerst erfolgreich agierte [41].

**Figure 4:**
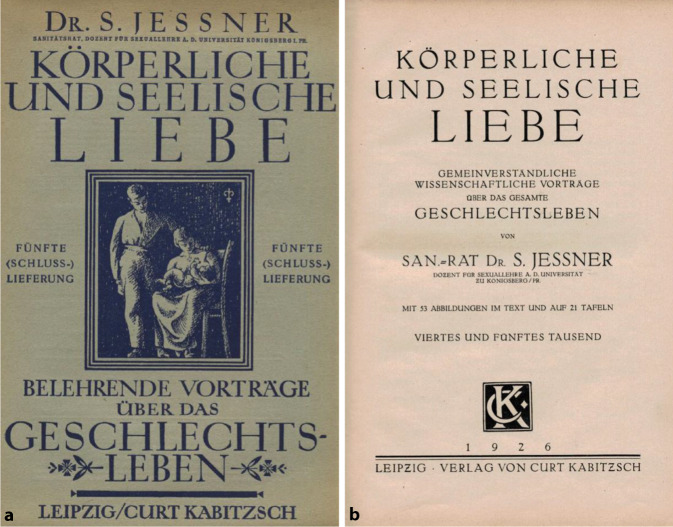

Ein ambivalentes Verhältnis zwischen Jessner und Hirschfeld lässt sich nicht zuletzt am Beispiel der Homosexualität illustrieren. Zwar traten beiden für eine Abschaffung des § 175 ein. Während dies für Hirschfeld jedoch Teil eines Prozesses der Depathologisierung und Normalisierung der Homosexualität sein sollte [42], hatte Jessner in seinem 1924 erschienenen Buch „Körperliche und seelische Liebe“ ([43]; Abb. 4) im Zusammenhang mit Homosexualität abwertend von

„*Mitläufer[n]“ gesprochen, „die aus Charakterschwäche, aus einem Mangel an Selbstbeherrschung, aus einem übergroßen Interesse für alles Pikante und Anrüchige, die sexuelle Lebensführung Perverser zeitweilig oder auch dauernd mitmachen, sich in den Strudel hineinziehen lassen, ohne von Hause aus irgendwie direkt dazu veranlagt, prädisponiert zu sein.“*[44].

Hirschfeld und die Wissenschaftler aus seinem engeren Umfeld, so Jessner weiter, hätten definitorisch *„nicht die Grenzen genug einzuhalten vermocht und die Kranken von den Mitläufern zu trennen verstanden.“*[44].

Im Übrigen – das war ihm offenkundig wichtig – litten die davon zu separierenden „wahren Homosexuellen“ Jessners Überzeugung zufolge an einem „Krankheitszustand“, an einer „Mißbildung“[45]. Deswegen und weil er sich u. a. daran störte, dass der Paragraph „den Homosexuellen eine Märtyrerkrone“ verschaffen würde [46], war er für dessen Abschaffung. Jessners Dualismus von ihrem Wesen nach homosexuellen Männern und solchen, die zu gleichgeschlechtlichen Handlungen „verführbar“ seien, war eine dominierende Position in der deutschen Medizin der Weimarer Republik und ebenso in der NS-Zeit [47, 48].

Diese Opposition zu Positionen und den sexualpolitischen Verbänden Hirschfelds schnitt Jessner jedoch nicht von allen Kontakten zu sexualwissenschaftlich und -medizinisch profilierten Kollegen ab: Albert Moll (1862–1939), der zweite „Protagonist der Berliner Sexualforschung“, häufig als Hirschfelds konservativer Gegenspieler beschrieben [49–51], fragte Samuel Jessner Mitte der 1920er-Jahre an, als es galt, einen Nachfolger für den konservativen Sexualethiker Seved Ribbing (1845–1921), der als der große Gegenspieler der Frauenrechtlerin Ellen Key (1849–1926) galt [52], für einen Beitrag über sexuelle Aufklärung für das „Handbuch der Sexualwissenschaft“ in der dritten Auflage von 1926 zu gewinnen [53].

## Fazit

Für die Bereiche Urologie und Dermatovenerologie können frühe Protagonisten des sich parallel entwickelnden Fachs Sexualmedizin durchaus nachgewiesen werden, darunter Carl Posner (1854–1928; [54]), Hermann Rohleder [55] und Samuel Jessner. Diese verfassten neben akademischen Schriften auch populäre Publikationen. Gerade der Vergleich von Rohleder und Jessner zeigt jedoch, dass neben Publikationstätigkeit auch andere Kriterien, wie der universitäre Standort oder die (Nicht‑)Mitgliedschaft in bestimmten Netzwerken der Sexualwissenschaft den Erfolg einer akademischen Positionierung mitbestimmen konnten. Samuel Jessners Erfolg, 1921 den ersten universitären Lehrauftrag für „Sexuallehren“ in Deutschland erhalten zu haben, ist zweifellos historisch.

Der vorliegende Beitrag zeigt allerdings, dass dafür keine besondere sexualwissenschaftliche Infrastruktur vor Ort ausschlaggebend gewesen ist. Der Dozent bewegte sich vielmehr zwischen allen Stühlen eines außeruniversitär aufblühenden Faches, suchte nur lose Anbindung an netzwerkartige Strukturen der Sexualreformbewegung und an das einflussreiche Institut für Sexualwissenschaft in Berlin. Vielmehr kann Jessners Erfolg dadurch erklärt werden, dass er den Kompromiss des „Weimar settlement on sexual politics“ personifizierte, der sexuellen Themen eine gewisse Relevanz einräumte, solange ihre öffentliche Präsenz nahezu unsichtbar blieb [56]. Gleichzeitig blieb sein Einfluss auf die deutschsprachige Sexualwissenschaft und Sexualmedizin – auch deshalb – überschaubar. Er starb am 7. Dezember 1929 in Königsberg^12^, als die Debatte um eine Reform des Sexualstrafrechts gerade hohe Welle schlug [57]. Nachrufe in sexualwissenschaftlichen Zeitschriften der Zeit sind keine überliefert. Spätestens mit der Machübernahme der Nationalsozialisten 1933 wurde die Erinnerung an ihn getilgt und verschüttet [58].

Es ist indes bis heute nicht gelungen im Bereich der Hochschulen eine gesicherte Stellung des Faches Sexualwissenschaft oder Sexualmedizin in Forschung und Lehre in Deutschland zu erreichen, eine Tatsache, die nach wie vor hochschulpolitisch relevant ist. Es gibt fast keine Lehrstühle für das Fach. Das Institut für Sexualwissenschaft der Universitätsklinik Frankfurt a. M., eine sexualpolitisch äußerst engagiert und international außerordentlich renommiert gewesene Einrichtung, wurde 2006 trotz massiven Protests, der auch aus der Ärzteschaft kam, abgewickelt [59]. Auf dem 121. Deutschen Ärztetag im Jahre 2018 in Erfurt wurde die Integration der Sexualmedizin in die (Muster‑)Weiterbildungsordnung der Bundesärztekammer beschlossen^13^, ein Musterkursbuch der Bundesärztekammer liegt seit dem Jahre 2020 für Weiterbildungskurse, die auch die DGU anbietet vor [61–63]. Ob diese Schritte mittel- oder langfristige eine Stärkung von Sexualwissenschaft/Medizin an den deutschen Universitäten oder Fachgesellschaften bedeuten, bleibt angesichts der in dieser Hinsicht skeptisch stimmenden Geschichte der Anerkennung dieses wichtigen Fachgebiets abzuwarten.

## Footnotes

1 *Dermatologische Zeitschrift* 1921 Personalien Sanitätsrat Dr. A. Jeßner in Königsberg hat einen Lehrauftrag für das Gesamtgebiet der Sexuallehre erhalten *Dermatologische Zeitschrift* 61 126.

2 Bisweilen abweichende Schreibweisen: Jesner und Jeßner.

3 Bienenstamm Hv 1841 Neue geographisch-statistische Beschreibung des kaiserlich-russischen Gouvernements Kurland, oder der ehemaligen Herzogthümer Kurland und Semgallen, mit dem Stifte Pilten. Reyer, Miltau und Leipzig.

4 Online stehen in der Berliner Staatbibliothek nur Adressbücher in diesem zeitlichen Abstand zur Verfügung: für das Jahr 1863, 1864, 1868 einziger Nameseintrag „Jessner“: Jessner H. M. Gesch Comm.1864: Raffineriegasse 4 (S 141), 1868: Vord. Vorst. 74. 75. (S 143).

5 Nürmberger C Th (1873) Adressbuch der Haupt- und Residenzstadt Königsberg1873. Aufgrund amtlicher Materialien und authentischer Privatnotizen redigiert, Nürmberger, Königsberg, S 73 (Hospitaldammgasse 4), ebenfalls 1876 unter gleicher Adresse S. 84.

6 Prof. für Dermatologie und ab 1931 Ordinarius in Breslau bis 1935, 1941 Flucht über Spanien und Portugal in die USA, 1970er-Jahre zurück in die Schweiz; in der Erinnerungskultur der internationalen Dermatologie über Eponyme wie Jessnersche Lösung/Peeling vgl. [18] oder [19].

7 Eine eingehende Darstellung im sexologischen Zusammenhang fehlt. Siehe einstweilen dazu insgesamt: [32].

8 HA Rep 76 Va Sekt 11 tit IV Nr 2 Bl 206 v.

9 Eintrag in: Deutscher Dermatologen-Kalender 1929. Via: WBIS.

10 GehPr Staatsarchiv IHARep76Va Sekt11 tit IVNr 20Bd13Bl208.

11 HARep76VaSekt11tit IVNr2Bl124v.

12 Geh Pr. Staatsarchiv VII HA B 42 Nr 3679 0002.

13 Zu Punkt III der Tagesordnung: [60].
